# Uptake of Primary Care Services and HIV and Syphilis Infection among Transgender Women attending the Tangerine Community Health Clinic, Bangkok, Thailand, 2016 – 2019

**DOI:** 10.1002/jia2.25683

**Published:** 2021-06-21

**Authors:** Frits van Griensven, Rena Janamnuaysook, Oranuch Nampaisan, Jitsupa Peelay, Kritima Samitpol, Stephen Mills, Tippawan Pankam, Reshmie Ramautarsing, Nipat Teeratakulpisarn, Praphan Phanuphak, Nittaya Phanuphak

**Affiliations:** ^1^ Institute of HIV Research and Innovation Bangkok Thailand; ^2^ Department of Epidemiology and Biostatistics University of California at San Francisco San Francisco CA USA; ^3^ Center of Excellence in Transgender Health Chulalongkorn University Bangkok Thailand; ^4^ Family Health International 360 Bangkok Thailand; ^5^ Thai Red Cross AIDS Research Centre Bangkok Thailand

**Keywords:** transgender women, primary care, epidemiology, HIV infection, sexually transmitted infections, syphilis, Bangkok, Thailand, Southeast Asia

## Abstract

**Introduction:**

Transgender women (TGW) need a specific package of primary care services usually not available in the publicly funded healthcare system. In addition, little is known about HIV and syphilis prevalence and incidence in clinic‐based samples of TGW. Here we evaluate the uptake of a transgender‐specific package of primary care services by TGW in Bangkok, Thailand and assess HIV and syphilis prevalence and incidence among them.

**Methods:**

Open cohort study of TGW attending services at the Tangerine Community Health Clinic from 2016 to 2019. Cross‐sectional and longitudinal analysis of routinely collected clinic data was performed to study trends in the number of clients, clinic visits and HIV and syphilis prevalence and incidence.

**Results:**

During the study period, 2947 TGW clients made a total of 5227 visits to Tangerine. The number of clients significantly increased from 446 in 2016 to 1050 in 2019 (*p* < 0.001) and the number of visits from 616 to 2198 during the same period (*p* < 0.001). Prevalence of HIV at first visit was 10.8% and of syphilis 9.8%. HIV incidence was 1.03 per 100 person years (PY) and of syphilis 2.06 per 100 PY of follow‐up. From 2016 to 2019, significant decreases occurred in the annual prevalence of HIV from 14.6% to 9.9% (*p *< 0.01). The annual prevalence of syphilis significantly increased from 6.6% in 2016 to 14.6% in 2018, and then decreased to 7.3% in 2019 (*p* < 0.001). The annual HIV incidence decreased during 2016 to 2019, from 1.68 to 1.28 per 100 PY, but this reduction was not statistically significant. The annual incidence of treponemal test seroconversion significantly increased from zero in 2016 to 4.55 per 100 PY in 2019 (*p* < 0.001).

**Conclusions:**

The increasing uptake of a transgender‐specific package of services, including co‐located gender affirmative hormone therapy, suggests this may be an effective model in engaging and retaining TGW in primary care. The decrease in HIV prevalence and low HIV incidence across calendar years point at a possible reduction of HIV acquisition among the TGW population served by Tangerine. The increasing prevalence of syphilis suggests ongoing high‐risk sexual behaviour and underscores the need for screening and treatment for this infection at the time of delivery of HIV services.

## 1 Introduction

The term transgender (TG) is commonly used as an umbrella concept to capture people who express discordance between their biological phenotype and social gender. Transgender women (TGW), here defined as persons assigned male sex at birth but who identify as women, need a specific array of healthcare services. This includes gender affirmative hormone therapy and other medical interventions. However, in most countries, including Thailand, such procedures are excluded from publicly and privately funded healthcare services. In addition, TGW face widespread stigma and discrimination both in the healthcare system and in society at large [1]. Because of these problems, TGW are at high risk for a variety of mental and physical health problems, including human immunodeficiency virus (HIV) and other sexually transmitted infections [2, 3]. In a global meta‐analysis of cross‐sectional studies conducted in 15 countries from 2000 to 2011, the pooled HIV prevalence among 11,066 TGW was 19.1% [4]. This meta‐analysis may have overestimated the HIV prevalence since most studies applied convenience sampling, which is likely to over‐represent high‐risk and street‐based TGW [5]. Presumably, the HIV prevalence in other segments of the TGW population, such as those not found at venues or those attending clinical services is lower.

In 2005, Thailand was the first country in Southeast Asia to formally begin integrated behavioural and biological HIV surveillance (IBBS) among TGW. Using venue‐day‐time sampling [6], the HIV prevalence among 474 TGW enrolled from Bangkok, Chiang Mai and Phuket in 2005 was 13.5% [7, 8]. In subsequent years, HIV prevalence fluctuated from 10% to 15% among women enrolled from these and other sites [9]. Following the initiation of IBBS, several studies have assessed the HIV prevalence among TGW attending HIV testing and counselling (HTC) services in public hospitals around Thailand. HIV prevalence among TGW attending these facilities ranged from 8.8% to 12.0% during 2011 to 2018 [10, 11, 12, 13]. Little information is available about HIV incidence among TGW in Thailand. One study reported an HIV incidence of 4.1 per 100 person years (PY) among TGW initially testing HIV‐uninfected but who seroconverted during follow‐up testing [13]. However, the number of seroconversions and the number of PY of follow‐up in this study were small (6/147.2 PY) [13]. Studies with respect to other sexually transmitted infections (STI) among TGW in Thailand are limited. One study evaluated point of care STI testing among 764 TGW enrolled in a Test and Treat cohort. Baseline prevalence of gonorrhoea and/or chlamydia in any compartment (oropharyngeal, anal, urethral) was 30.4%, and the incidence of these infections during follow‐up, 23.7 per 100 PY. No studies could be identified about the prevalence and incidence of syphilis infection among TGW in Thailand.

To start addressing the specific healthcare needs of transgender people and to improve access and retention in primary care, the Tangerine Community Health Clinic was founded in 2015. Tangerine provides a comprehensive package of primary care services, including HIV and STI testing, treatment and prevention services. The package further includes gender affirmative hormone therapy, sexual identity counselling and other transgender‐specific services.

To evaluate the uptake of services and to obtain more information about HIV and STI prevalence and incidence among TGW we analysed routine data collected at Tangerine. This report describes trends in service uptake, and in HIV and syphilis prevalence and incidence seen among TGW clients from 2016 to 2019.

## 2 Methods

### 2.1 Venue and services

The goal of Tangerine is to engage and retain transgender people in primary care. The clinic supports an enabling environment and staff are receptive to the specific health needs and concerns of transgender people. Members of the transgender community play pivotal roles in the management and daily running of the clinic. A comprehensive package of services is offered, including gender affirmative hormone therapy, access to HIV and STI testing and treatment, HIV pre‐ and post‐exposure prophylaxis, evaluation for anogenital cancer and other malignancies, vaccination and gender‐identity and risk‐behaviour counselling, including free access to condoms and lubricants. HIV and STI testing services, pre‐ and post‐exposure prophylaxis, same‐day antiretroviral treatment (if found HIV‐infected, irrespective of CD4+ cell count) and counselling are provided at no cost to clients.

### 2.2 Population

Our study population consists of TGW clients who accessed services at the Tangerine Community Health Clinic. At first visit and during six‐month follow‐up appointments, clients were offered HIV (if found HIV‐uninfected at baseline or at follow‐up visits) and syphilis testing. Those testing HIV‐infected and those with active syphilis infection received treatment according to Thai national guidelines.

### 2.3 Study period

Tangerine started its service delivery in late 2015, when four new clients were seen. Because of their small number, these individuals were not included in our analysis and January 1, 2016, was taken as the left cut‐off date. December 31, 2019 was taken as the right cut‐off date.

### 2.4 Laboratory methods

Blood samples were tested for the presence of HIV with Alinity i HIV Ag/Ab Combo (Abbott Ltd., Wiesbaden, Germany) or HISCL HIV Ag + Ab Assay Kit (Sysmex, Kobe, Japan), and if found reactive, confirmed with Diagnostic Kit for HIV (1 + 2) Antibody (Colloidal Gold) V2 (Shanghai Kehua Bio‐Engineering Co., Ltd., Shanghai, China) and ADVIA Centaur CP HIV Ready Pack (Siemens, New York, USA) according to the Thai national rapid HIV testing algorithm. History of syphilis (here defined as the presence of antibodies against *Treponema pallidum* (TP)) was evaluated with Alinity i Syphilis TP Reagent Kit (Abbott Ltd., Wiesbaden, Germany). For diagnostic purposes, reactive samples were further evaluated with a Plasmatec Rapid Plasma Reagin test (Lab21 Healthcare Ltd., Dorset, England) when indicated.

### 2.5 Statistics

Statistical analysis was conducted with Stata version 14.1 (Statcorp, College Station, TX, USA). HIV and syphilis prevalence were calculated at the first visit when this testing was performed. HIV incidence and incidence of treponemal test seroconversion were computed among those who tested non‐reactive for HIV or treponemal test at first visit, but who returned for retesting later. Times from the date of the first non‐reactive test to the latest non‐reactive test or to the midpoint between the dates of the last non‐reactive and first reactive HIV or treponemal test were used to calculate PY of follow‐up time. Trends in the number of clients, visits and HIV prevalence and prevalence of past or current syphilis infection were evaluated for statistical significance using chi‐square. Trends in HIV incidence and incidence of treponemal test seroconversion were assessed using Poisson regression testing.

### 2.6 Ethical review

The institutional review board (IRB) of the Faculty of Medicine of Chulalongkorn University, Bangkok, Thailand (IRB00001607), reviewed and approved of the protocol describing the service delivery model and the collection, storage and analysis of data. The Chulalongkorn IRB is the ethical review committee of record to which Tangerine defers for review and approval of programme and research activities performed at its clinic.

## 3 Results

During the 2016 to 2019 period, 2943 TGW clients made a total of 5227 visits to Tangerine (Table 1). The number of clients significantly increased from 446 in 2016 to 1050 in 2019 (*p* < 0.001) and the number of visits from 616 to 2198 during the same period (*p* < 0.001). Of clients, 69.5% (2046/2943) paid one visit, 15.1% (444/2943) paid two visits and 15.4% (453/2943) paid three visits or more to the clinic. Client’s median age was 25 (interquartile range 22 to 29 years) and slightly more than half (50.6% or 1488/2943)) of clients were in the 22 to 29 years old age category. Most visits (54.9% or 2872/5227) were in this age category as well. Close to all (96.6% or 2842/2943) were of Thai nationality and 72.1% (2011/2793) lived in the Bangkok metropolitan area. More than half (65.9% or 1939/2943) had been tested for HIV infection previously and 1.5% (44/2943) had a known prior HIV reactive test. Prevalence of HIV at first visit during 2016 to 2019 was 10.8% (319/2943) and of past or current syphilis 9.8% (218/2223). Overall HIV incidence was 1.03 per 100 PY and that of treponemal test seroconversion, 2.06 per 100 PY (Table 1).

**Table 1:** Prevalence of HIV and past or current syphilis and incidence of HIV and treponemal test seroconversion among transgender women, by calendar year and age group – Tangerine Community Health Clinic, Bangkok, Thailand, 2016 to 2019

Characteristic			Prevalence		Incidence			
	Clients	Visits	HIV	Syphilis, past or current	HIV		Treponemal test seroconversion	
					Incidence density^a^		Incidence density^a^	
	N (%)	n (%)	n/N (%)	n/N (%)	n/PY	95% CI	n/PY	95% CI
Calendar year								
2016	446 (15.2)	616 (11.8%)	65/446 (14.6)	25/381 (6.6)	2/119.24	1.68 (0.20 to 6.06)	0/120.20	0 (0 to 3.07)
2017	448 (15.2)	767 (14.7)	36/448 (8.0)	42/321 (13.1)	0/249.56	0 (0 to 1.48)	2/248.98	0.80 (0.10 to 2.90)
2018	999 (34.0)	1646 (31.4)	114/999 (11.4)	80/549 (14.6)	5/409.64	1.22 (0.40 to 2.85)	4/398.32	1.00 (0.27 to 2.57)
2019	1050 (35.6)	2198 (42.1)	104/1050 (9.0)	71/972 (7.3)	5/391.53	1.28 (0.41 to 2.98)	18/395.36	4.55 (2.70 to 7.20)
*P* values	<0.001	<0.001	0.01	<0.001		0.55		<0.001
Age (years)^b^								
≤21	734 (24.9)	1202 (23.0)	60/734 (8.2)	32/538 (5.9)	4/262.48	1.52 (0.42 to 3.90)	3/256.29	1.17 (0.24 to 3.42)
22 to 29	1488 (50.6)	2872 (54.9)	158/1488 (10.6)	115/1148 (10.0)	6/694.64	0.86 (0.32 to 1.88)	16/683.48	2.34 (1.34 to 3.80)
≥30	721 (24.5)	1153 (22.1)	101/721 (14.0)	71/537 (13.2)	2/212.84	0.94 (0.11 to 3.39)	5/213.09	2.35 (0.76 to 5.48)
*P* values	<0.001	<0.001	0.002	<0.001		0.50		0.35
Overall	2943	5227	319/2943 (10.8)	218/2223 (9.8)	12/1169.97	1.03 (0.53 to 1.79)	24/1162.86	2.06 (1.32 to 3.07)

CI, confidence interval; PY, person year.

^a^ Per 100 person years.

^b^ Age at time of first visit.

There was an association between prevalent past or current syphilis and HIV infection. Among persons testing treponemal test reactive, the HIV prevalence was 33.5% (73/218), whereas among those testing non‐reactive it was 8.6% (173/2005) (Risk Ratio = 3.86 and 95% confidence interval [3.05, 4.89]). The overall prevalence of HIV and past or current syphilis co‐infection was 3.3% (73/2223) (Table 2).

**Table 2:** Prevalence of HIV and syphilis co‐infection among transgender women — Tangerine Community Health Clinic Bangkok, Thailand, 2016 to 2019

HIV			
Syphilis^a^	Reactive n (%)	Non‐reactive n (%)	Total n (%)
Reactive	73 (33.5)	145 (66.5)	218 (100)
Non‐reactive	173 (8.6)	1832 (91.4)	2005 (100)
Total	246 (11.1)	1977 (88.9)	2223 (100)

^a^ Past or current syphilis based on the presence of antibodies against *Treponema pallidum* (TP).

From 2016 to 2019, significant decreases occurred in the annual prevalence of HIV from 14.6% (65/446) to 9.9% (105/1050) (*p* < 0.01). The annual prevalence of past or current syphilis significantly increased from 6.6% (25/381) in 2016 to 14.6% (80/594) in 2018, and then decreased to 7.3% (71/927) in 2019 (*p* < 0.001). Although overall HIV prevalence was significantly higher among older clients (14.0% or 101/721) for those aged ≥30 years versus 8.2% or 60/734 for those aged ≤21 years, *p* < 0.002), overall HIV incidence was slightly higher in the younger age group (1.52 per 100 PY for those aged ≤21 years versus 0.94 per 100 PY for those aged ≥30 years). The latter difference was not statistically significant (Table 1). The annual HIV incidence decreased during 2016 to 2019, from 1.68 to 1.28 per 100 PY (not significant). The annual incidence of treponemal test seroconversion significantly increased from zero in 2016 to 4.55 per 100 PY in 2019 (*p* < 0.001). There was no statistically significant difference in the overall HIV incidence and incidence of treponemal test seroconversion by age category (Table 1).

## 4 Discussion

Our analysis showed an increasing temporal trend in the number of TGW clients and visits to the Tangerine Community Health Clinic from 2016 to 2019. Laboratory testing data showed a decreasing trend of the annual HIV prevalence during the same time period. The annual prevalence of past or current syphilis infection displayed an inverted U‐shape with a decline during 2019. Both HIV and past or current syphilis prevalence significantly increased with age. The overall incidence of HIV infection during follow‐up was 1.03 per 100 PY and that of treponemal test seroconversion, 2.06 per 100 PY. While the annual HIV incidence did not change significantly over time, an increasing trend in the annual new treponemal test seroconversions was found. There were no differences in HIV incidence and incidence of treponemal test seroconversion by age.

The increasing annual number of clients and clinic visits to Tangerine are indicative of the demand for transgender‐specific primary care services and the success of the Tangerine model in delivering them. This observation confirms earlier notions regarding the disconnect between existing primary care services and the healthcare needs of transgender people [14]. Also, our data corroborate earlier notions of a lower HIV prevalence among TGW attending clinical care services as compared to women recruited from entertainment or street‐based venues [4, 5].

The decrease in the annual HIV prevalence combined with low annual HIV incidence across calendar years suggests a reduction in HIV acquisition among the TGW population served by Tangerine. While the annual HIV prevalence may also decrease as a result of persons moving out of the population due to HIV‐related morbidity and mortality, this is unlikely in the Thai context. Thailand introduced publicly funded universal access to antiretroviral treatment (ART) for all HIV infected Thais regardless of CD4+ cell count in 2014 [15]. Currently, it is estimated that approximately 75% of the HIV‐infected population is on ART [13], and in some studies close to 90% of MSM and TGW identified as HIV infected were accessing treatment [12, 16].

However, the cumulative HIV prevalence in the reference population may be higher since those testing HIV‐infected previously are not included in prevalence calculations during subsequent years.

In stark contrast with our findings, HIV prevalence among street‐ and venue‐based TGW enrolled in IBBS in Bangkok increased from 9.9% in 2014 to 17.3% in 2018 [17]. This confirms earlier suggestions that the risk for HIV infection in the latter group may be elevated compared to TGW accessing clinical services [5], such as those delivered by Tangerine. The decreasing HIV prevalence seen among Tangerine clients may also result from a reduction in new HIV infections associated with the recent scaling of HIV treatment for prevention and HIV pre‐exposure prophylaxis (PrEP) for MSM and TGW in Thailand [11, 12, 13, 18, 19, 20, 21]. Promotion and providing access to HIV treatment and prevention services for TGW is one of the main goals of Tangerine.

While a rising epidemic of syphilis infection had already been reported among MSM in Bangkok [22, 23], our data provide evidence for the existence of a similar epidemic among TGW in the Thai capital. Reemerging and increasing epidemics of syphilis among MSM and TGW have been reported from around the world [24, 25, 26]. Generally, the rise in syphilis and other STI among MSM and TGW has been attributed to an increase in unprotected sexual behaviour associated with “treatment optimism”, following the introduction of highly active antiretroviral treatment during the mid‐nineties of the past century. And more recently as a result of HIV treatment for prevention and HIV PrEP. The use of antiretroviral drugs for prevention provides protection against HIV transmission but not against other STI, such as syphilis. Aside from its own intrinsic pathogenic properties, syphilis is also an established risk factor for HIV transmission among MSM and TGW [27, 28]. This association was also shown in our data, where those with a reactive syphilis antibody test at baseline were almost four times more likely to test HIV infected as well. Infection with syphilis may be asymptomatic and go unnoticed for several years until open expression of disease. Screening and treatment for syphilis and other STI at time of delivery of HIV testing, treatment and prevention services is therefore recommended.

Even though HIV and syphilis prevalence significantly increased with increasing age, there was no effect of age on HIV and treponemal test seroconversion incidence. Age is a proxy for duration of exposure, which is reflected in higher HIV and syphilis prevalence among those older. Since HIV and treponemal test seroconversion incidence were found independent of age, prevention efforts should target all age groups.

Only a few of our clients testing HIV positive were aware of their HIV infection. This finding underscores the need for increased access and more frequent HIV testing, especially for key populations, such as TGW. Knowledge of HIV status is necessary for linkage to care, treatment and prevention services, including immediate initiation of ART and HIV PrEP. In the context of recent advances in biomedical HIV prevention, HIV testing in clinic‐based settings, such as Tangerine, may play an important role in further reducing the spread of HIV infection among TGW and their sexual partners.

Although all Tangerine clients were tested for HIV and most for syphilis at their first visit, re‐testing for these infections during follow‐up was less frequent. Despite Tangerine recommending yearly HIV and syphilis re‐testing, several clients choose not to do so. To some extent this is inherent to the open cohort character of the underlying service paradigm of Tangerine, in which clients can come in, return and get re‐tested as they see fit. Nevertheless, this observation leaves room for improvement. Approaches being considered are the introduction of provider‐initiated opt‐out HIV testing (as opposed to client‐initiated HIV testing) [29, 30] and rapid dual testing for HIV and syphilis infection. Another avenue to accomplish more frequent HIV testing is the delivery of HIV PrEP. To increase HIV testing and PrEP uptake among TGW, the Institute of HIV Research and Innovation, of which Tangerine is a part, recently initiated the “PrEP in the City” project, a dedicated PrEP campaign for TGW in Thailand [31]. While increased evaluation for HIV and syphilis may not change their incidence, it certainly would increase the number of TGW clients with correct knowledge of their current infection status. US CDC and WHO guidelines recommend that individuals at risk are screened for HIV infection at least annually and, in some situations, every three to six months [29, 30]. In addition, guidelines suggest that all persons who seek evaluation and treatment for STI should be screened for HIV infection and vice versa [32]. This is believed to enable those who have tested HIV negative previously to stay HIV negative and identify those who have become HIV positive as early as possible for treatment and prevention of onward transmission [33].

Our study also had some limitations. TGW clients who attended services at Tangerine are self‐selected and may not be representative for the TGW community in Bangkok at large. Most likely their attendance has been motivated by the key population‐led nature of the clinic and the specific TGW services it provides [34]. These characteristics may attract women of certain socio‐economic and relational backgrounds and possibly higher levels of risk consciousness. These properties could be different in women not attending such clinical services. This may artificially bias our estimates of HIV and syphilis prevalence and incidence downwards. Indeed, the HIV prevalence among TGW recruited in IBBS from street‐ and entertainment‐based venues around Bangkok was found much higher and increasing. On the other hand, if self‐selection bias is consistent over time, it might not affect the validity of our trend analysis, showing decreases in the annual HIV and syphilis prevalence across calendar years. Finally, our study may have underestimated the incidence of syphilis. Our definition of new syphilis infection was based on seroconversion for the presence of antibodies against *Treponema pallidum*, the aetiological agent of syphilis. This definition does not include possible cases of syphilis re‐infection among those who were previously found TPHA reactive. Unlike HIV, syphilis is a treatable bacterial infection after which re‐infection is possible.

## 5 Conclusions

Our analysis of routine data collected at the Tangerine Community Health Clinic shows increasing uptake of its service model by the TGW community in Bangkok. The number of clients almost tripled and the number of visits increased more than fourfold from 2016 to 2019. The relatively low annual HIV incidence and decreasing the annual HIV prevalence may be signs of a declining HIV epidemic in our clinic‐based sample of TGW. Results from concurrent testing for syphilis infection suggest ongoing high‐risk sexual behaviour and underscores the need for screening and treatment for this infection at the time of delivery of HIV services. Altogether, these data suggest that Tangerine’s comprehensive package of services, including co‐located gender affirmative hormone therapy, may be an effective model to engage and retain transgender people in primary care.

## Competing Interests

The authors do not report any conflicts of interest.

## Authors’ Contributions

FVG, PP and NP conceived the primary care delivery model applied at Tangerine; KS, NT, NP, RJ and RR conducted and oversaw the implementation of clinical services and collection of data; JP and ON conducted data‐management and statistical analysis; TP performed laboratory testing; SM assisted with project management; FVG drafted the initial paper; all authors reviewed, provided feedback and approved of the final manuscript.
